# Gene expression in histologically normal epithelium from breast cancer patients and from cancer-free prophylactic mastectomy patients shares a similar profile

**DOI:** 10.1038/sj.bjc.6605576

**Published:** 2010-03-02

**Authors:** K Graham, A de las Morenas, A Tripathi, C King, M Kavanah, J Mendez, M Stone, J Slama, M Miller, G Antoine, H Willers, P Sebastiani, C L Rosenberg

**Affiliations:** 1 Genetics and Genomics Program, Boston University School of Medicine and Boston Medical Center, Boston, MA, USA; 2 Department of Pathology, Boston University School of Medicine and Boston Medical Center, Boston, MA, USA; 3 Department of Medicine, Boston University School of Medicine and Boston Medical Center, Boston, MA, USA; 4 Department of Surgery, Boston University School of Medicine and Boston Medical Center, Boston, MA, USA; 5 Department of Radiation Oncology, Boston University School of Medicine and Boston Medical Center, Boston, MA, USA; 6 Department of Biostatistics, Boston University School of Public Health, Boston, MA 02118, USA

**Keywords:** breast cancer, gene expression, histologically normal epithelium, prophylactic mastectomy, risk

## Abstract

**Introduction:**

We hypothesised that gene expression in histologically normal (HN) epithelium (NlEpi) would differ between breast cancer patients and usual-risk controls undergoing reduction mammoplasty (RM), and that gene expression in NlEpi from cancer-free prophylactic mastectomy (PM) samples from high-risk women would resemble HN gene expression.

**Methods:**

We analysed gene expression in 73 NlEpi samples microdissected from frozen tissue. In 42 samples, we used microarrays to compare gene expression between 18 RM patients and 18 age-matched HN (9 oestrogen receptor (ER)+, 9 ER−) and 6 PM patients. Data were analysed using a Bayesian approach (BADGE), and validated with quantitative real-time PCR (qPCR) in 31 independent NlEpi samples from 8 RM, 17 HN, and 6 PM patients.

**Results:**

A total of 98 probe sets (86 genes) were differentially expressed between RM and HN samples. Performing hierarchical analysis with these 98 probe sets, PM and HN samples clustered together, away from RM samples. qPCR validation of independent samples was high (84%) and uniform in RM compared with HN patients, and lower (58%), but more heterogeneous, in RM compared with PM patients. The 86 genes were implicated in many processes including transcription and the MAPK pathway.

**Conclusion:**

Gene expression differs between the NlEpi of breast cancer cases and controls. The profile of cancer cases can be discerned in high-risk NlEpi from cancer-free breasts. This suggests that the profile is not an effect of the tumour, but may mark increased risk and reveal the earliest genomic changes of breast cancer.

The earliest recognised breast cancers (*in situ* malignancies) already harbour many of the genomic aberrations that are characteristic of invasive disease (Allred *et al*, 2008; Tamimi *et al*, 2008; Kuerer *et al*, 2009). Attention has focused recently on alterations in earlier cancer precursors, including hyperplastic lesions and even histologically normal (HN) epithelium (reviewed in Simpson *et al*, 2005; Mastracci *et al*, 2007; Heaphy *et al*, 2009). However, there remains a gap in the current knowledge of the genomic features of breast cancer precursors. Investigating these precursors should elucidate important steps in breast cancer initiation and early progression (Thompson *et al*, 2008).

Many studies have used microarrays to investigate breast cancer gene expression (for recent reviews see Bao and Davidson, 2008; Sotiriou and Pusztai, 2009). Early studies led to the identification of major breast cancer subtypes: Luminal A, Luminal B, normal breast-like, ERBB2, and Basal (Perou *et al*, 2000; Sorlie *et al*, 2001). Subsequent work refined and validated the gene expression signatures of subtypes (Sorlie *et al*, 2003; Sotiriou *et al*, 2003; Calza *et al*, 2006; Kapp *et al*, 2006) and developed signatures to prognosticate patient survival and predict response to therapies (van de Vijver *et al*, 2002; van ‘t Veer *et al*, 2002; Paik *et al*, 2004; Ma *et al*, 2006).

Much less is known about the gene expression in premalignant breast tissue, and studies that focused on HN epithelium (NlEpi) are particularly limited (Finak *et al*, 2006; Grigoriadis *et al*, 2006; Tripathi *et al*, 2008; Chen *et al*, 2009). This is partly because of the difficulty in obtaining homogeneous epithelial cell populations from fresh tissue. Knowledge of gene expression changes in these tissues could generate novel tools to integrate into existing risk-assessment models (e.g. the Gail model (Gail *et al*, 1989, 2007)) to improve their accuracy. Some evidence already exists suggesting that alterations could have clinical significance (Yang *et al*, 2005; Chen *et al*, 2009). In our pilot study, using a different statistical approach and study design, we identified the differences in gene expression between microdissected NlEpi of controls (women undergoing reduction mammoplasty (RM)) and those of cases (women with oestrogen receptor (ER)+ breast cancers) (Tripathi *et al*, 2008). That study, however, could not address whether these differences were an effect of the existing tumour, a marker of increased cancer risk, or a profile of early carcinogenesis.

These results led us to hypothesise that altered gene expression in the NlEpi of breast cancer patients may be a generaliseable finding that occurs in both ER− and ER+ cases. Further, we speculated that altered gene expression would be discerned in the NlEpi of cancer-free breasts from some women at high breast cancer risk, and would resemble gene expression of breast cancer patients. If this were true, then the expression changes would not be an effect of the tumour, but instead may be a marker of increased breast cancer risk, or an indication of breast cancer's earliest gene expression changes. Understanding these early alterations may help create new prevention agents and risk-assessment tools.

To test our hypothesis, we compared gene expression in 73 NlEpi samples microdissected from snap-frozen primary tissues from three groups of women: (1) women at usual breast cancer risk undergoing mammoplasty reduction (RM); (2) women with breast cancer undergoing surgery for either an ER+ or ER− breast tumour (HN); this group was tightly age matched to the RM; and (3) high-risk patients, consisting of women undergoing prophylactic mastectomy (PM) of a cancer-free breast because of cancer in the contralateral breast, with a strong family history of breast cancer, or positive status as a *BRCA* mutation carrier.

## Materials and methods

### Breast tissue acquisition and sample preparation

**Figure 1:**
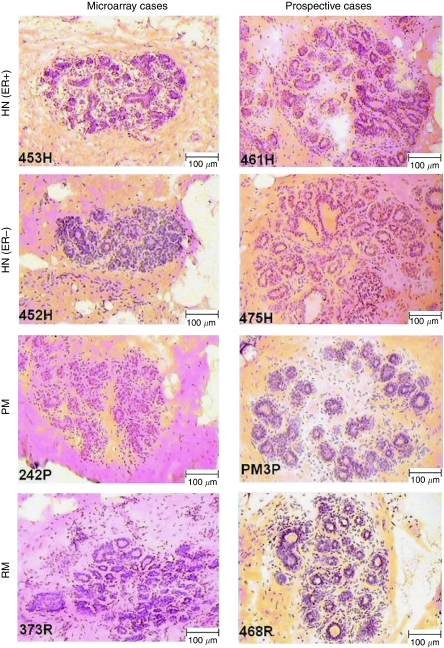
Representative histologically normal epithelium. Representative examples of 10 *μ*m H&E-stained guide sections showing histologically normal epithelial cells identified for microdissection.

All samples were obtained using an IRB-approved protocol for collection of de-identified breast tissue not required for histological diagnosis. Tissue preparation, microdissection, RNA extraction and amplification, array hybridisation, and data normalisation were performed as described earlier (Tripathi *et al*, 2008). Briefly, tissues were snap frozen, embedded in optimal cutting temperature embedding medium, sectioned at 10 *μ*m, stained with diluted haematoxylin and eosin (H&E) (see Figure 1), and then NlEpi – both TDLUs and ducts – was microdissected (see Supplementary Figure S1). Most HN samples were ‘tumour-adjacent’ (i.e. located 1–2 cm from the tumour) on blocks lacking malignant cells. Some HN lay further away, but still in the same quadrant as the tumour, as most surgeries were lumpectomies. Great care was taken to avoid microdissecting any proliferative cells, even simple hyperplastic lesions. The RNA was extracted from microdissected cells using the PicoPure extraction kit (Molecular Devices, Sunnyvale, CA, USA). For samples undergoing microarray, RNA was amplified after extraction and gene expression was measured using the HU133A chip (Affymetrix, Santa Clara, CA, USA), a technique that yields reliable and reproducible results (King *et al*, 2005). cel files were processed with MAS 5.0 using standard procedures for quality control, and normalisation was limited to re-scaling each sample to a mean intensity of 200. The microarray data from these samples are available from the NCBI Gene Expression Omnibus (http://www.ncbi.nlm.nih.gov/geo) under accession GSE20437.

### Patient groups

Gene expression in NlEpi was examined by microarray in 42 samples (see Table 1). The primary analysis was between two groups: the control group of women undergoing RM who lacked any personal or strong family history of breast cancer and whose resected breast tissue was diagnosed as benign (RM, *n*=18); and the case group of women with breast cancer undergoing surgery for their cancer, who had not undergone chemotherapy or radiation treatment before tissue acquisition (HN, *n*=18, 9 ER+, 9 ER−). Controls and cases were tightly age matched – no pair differed by more than 2 years – to adjust for age-associated changes and generate age-independent data. Data from 11 RM and 5 HN samples were reported earlier (Tripathi *et al*, 2008). The third, the high-risk group, consisted of women with a personal history or with a strong family history of breast cancer, or who were known BRCA mutation carriers, who were undergoing PM of a cancer-free breast (PM=6). If a patient had a personal breast cancer history, tissue was obtained from the uninvolved breast. The ages of PM cases fell within the range of the RM and HN groups.

Gene expression was examined by quantitative real-time PCR (qPCR) in a prospective validation of NlEpi in an independent set of 31 samples, defined using the same criteria as above. These samples included 8 RM, 17 HN (9 ER+, 8 ER−), and 6 PM cases (Supplementary Table S1).

### Identification of differentially expressed genes between RM and HN

A total of 9321 probe sets with <20% detectable hybridisation were removed, leaving 12 962 probe sets for analysis. Gene expression data of the probe sets that passed quality control filters were analysed by principal component analysis, and by a robust version of Bayesian Analysis of Differential Gene Expression (BADGE) (Sebastiani *et al*, 2006) to identify genes that were differentially expressed between RM and HN. The BADGE uses a model-averaging approach to identify probe sets with a different expression in two biological conditions and scores the evidence of differential expression by the probability that the fold change of expression is >1 or <1. The *P*-value is then calculated as 1 minus this probability, so that the smaller the *P*-value the stronger the evidence of differential expression. The method has a very large sensitivity but low specificity with small sample sizes – we showed (Sebastiani *et al*, 2006) that with 20 samples per group, the sensitivity to detect a fold change of 2 or larger can be 100%, but specificity can be <70%. Therefore, to reduce the chance of false positives, we used an extrinsic leave-one-out cross-validation implemented in BADGE to select those probe sets that showed robust changes of expression between groups (Singh *et al*, 2002). The leave-one-out cross-validation consisted of removing one case at a time from the data set and using the remaining samples to detect the probe sets with differential expression with a false discovery rate <6%. This threshold on the false discovery rate was chosen to trade off sensitivity and specificity. Probe sets selected at least 80% of the time were included in the final list of differentially expressed genes and the final *P*-values and fold changes are based on all samples (Table 2). For robustness, *P*-values from the traditional *t*-test of log-transformed gene expression data were also calculated. Heat maps were generated using the package HeatPlus from Bioconductor and simple hierarchical clustering was used to cluster samples on the basis of their expression profiles. To test the likelihood that the same cluster results are obtained by chance, we performed a Monte Carlo simulation in which we randomly permuted the sample labels 10 000 times, and computed the frequency of cluster results matching the observed one.

### Gene expression in PM samples

The expression data of the 98 probes selected in the comparison of HN and RM samples were examined in the six PM samples. We examined the Spearman correlation of fold change between RM and HN samples, and between RM and PM samples, and then performed hierarchical clustering to examine how PM samples cluster relative to RM and HN samples. We conducted a similar Monte Carlo simulation to test the likelihood of observing cluster results by chance.

### Validation of microarray data through qPCR

Genes were selected for validation on the basis of consistent expression among samples in the RM and HN groups, a strongly significant *P*-value, and biological relevance to cancer. On the basis of these criteria, six test genes were selected: AHNAK, ATF3, BTG2, CLU, EGR1, and FOS. We also selected an endogenous control gene, CPSF6, with consistent expression between groups. For each gene, we selected intron-spanning TaqMan primers (ABI, Foster City, CA, USA) that overlapped with the Affy probe set target on the HU-133A chip and generated amplicons <110 nucleotides.

For each qPCR reaction, 2 ng of unamplified RNA was reverse transcribed using random hexamers with the TaqMan Multiscript RT reagent kit (ABI). For each sample, the dCT value for each test gene was calculated by subtracting the CT value of the reference gene, CPSF6, from the CT value of the test gene. For each group (RM, HN, and PM), each test gene's mean dCT value and the standard error of the mean were calculated using the data analysis package in Microsoft Excel. To assess validation for each test gene, the dCT value of each HN and PM sample was compared with the mean RM dCT value. For each comparison, validation was defined as expression in the direction predicted by microarray analysis. The mean ddCT values for each gene were calculated by subtracting the mean dCT value of the reference group (RM) from the mean dCT value of each test group (HN or PM). Data were plotted as 2^−ddCT^ (i.e. mean fold change) using Graph Pad Prism software. To determine whether dCTs differed between groups (RM *vs* HN and RM *vs* PM), we used a one-tailed two-sample *t*-test assuming equal variances to compare the dCT values for individual samples between groups.

### Annotation and analysis of differentially expressed genes

The 98 probe-set list was uploaded into DAVID (http://david.abcc.ncifcrf.gov/home.jsp) and analysed using the functional annotation enrichment analysis to determine overrepresented GO terms and PANTHER functions. Gene set enrichment analysis (GSEA) (Mootha *et al*, 2003; Subramanian *et al*, 2005) and ingenuity pathway analysis (http://www.ingenuity.com) were also used to identify biological functions, canonical pathways, and functional gene classification terms overrepresented in the 98 probe sets.

## Results

### Gene expression by microarray in RM and HN samples

Table 1 presents summary information for the 42 RM, HN, and PM samples investigated by microarray analysis. The initial analysis was conducted between the RM group (*n*=18, mean age=51.4 years) and the HN group (*n*=18; nine from patients with ER+ tumours and nine from patients with ER− tumours, mean age 52.8 years). The RM and HN samples were directly age matched by not more than 2 years, as age is known to influence gene expression (Yau *et al*, 2007; Anders *et al*, 2008; Euhus *et al*, 2008). Thus, this comparison generates an age-independent gene expression profile.

To determine how well the 18 RM and 18 HN samples separated using the entire microarray data set, we performed a principal component analysis. The results indicated that these groups may be distinguishable – a fraction of the RMs (5 out of 18) clustered together away from the other samples (see Supplementary Figure S3). Next, we used BADGE to identify 98 probe sets (reflecting 88 independent transcripts or 86 identified genes) that were significantly differentially expressed between groups (see Table 2). Of the 98 probe sets, 66 (67%) had a higher gene expression in RM samples, and 32 of the 98 (33%) had a higher gene expression in HN samples. In all, 36 of the 98 probe sets (37%) were among the 127 probe sets identified in our initial report, which compared NlEpi between RM and HN samples only from ER+ breast cancer patients (Tripathi *et al*, 2008). A clustering analysis of RM and HN samples using these 98 probe sets shows that the two groups generally separate, although five HN samples cluster with RMs (see Supplementary Figure S2). In the Monte Carlo procedure, only 1 in 10 000 simulations produced the same cluster result, hence the probability that our results are due to chance is minimal. We reviewed the clinical and pathological features of these HN samples (patient age and tumour grade, ER and human epidermal growth factor receptor-2 (HER2) expression status, lymph node involvement, and NlEpi's distance from tumour). These features did not differ between clusters, although 80% of the HN samples that clustered with RMs were ER+ (4 out of 5) and 100% were HER2− (4 out of 4), whereas only 38% of the remaining HN samples were ER+ (5 out of 13) and only 42% were HER2− (5 out of 12). This trend is suggestive, but the differences were not statistically significant. In contrast, we did find a difference between the two groups of RM samples, using the only clinico-pathological feature of the RM samples that could be evaluated, which was age. The eight RMs clustering with the HNs were older than the remaining 10 RMs: mean ages were 56 years (range 44–75) *vs* 48 years (range 36–60) (*P*=0.04).

### Gene expression using qPCR in RM and HN samples

**Figure 2:**
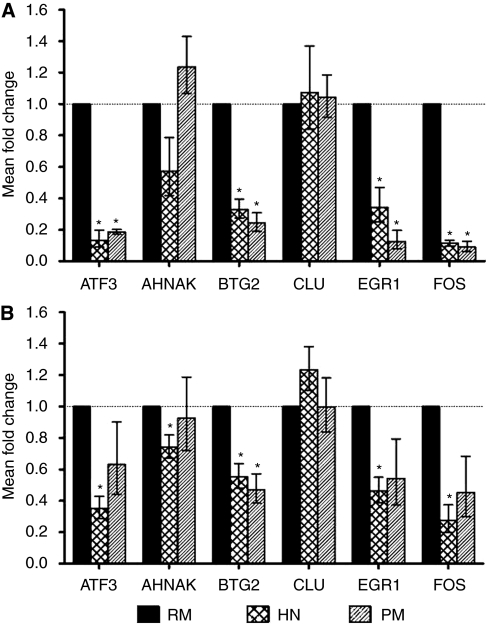
qPCR validation of microarray data. (**A**) depicts results using RNA from original samples (technical validation) and (**B**) depicts results using RNA from independent samples (prospective validation). Each panel shows qPCR results for six test genes. Test genes are listed on the *x* axis, and the mean fold change in expression in each sample group (HN or PM), compared with expression in the reference group (RM), is shown on the *y* axis. Fold changes were calculated using the ddCT method, in which fold change data are represented as 2^−ddCT^. Error bars depict the standard error of the mean dCT values. Significant differences (*P*<0.05) are denoted with an asterisk.

We used qPCR to validate the microarray-generated gene expression data. First, we examined unamplified RNA remaining from samples used for microarray (technical validation, see Figure 2A), and then we examined unamplified RNA from independent samples (prospective validation, see Figure 2B). We selected six test primers (AHNAK, ATF3, BTG2, CLU, EGR1, and FOS) and one endogenous control primer (CPSF6). For the technical validations, unamplified RNA from 24 of the original 36 samples was available (13 HN and 11 RM). Three to seven HN samples were tested with each primer and compared with four to seven RM samples. Overall, 27 out of 31 (87%) reactions validated the microarray data. Each of five primers (AHNAK, ATF3, BTG2, EGR1, and FOS) validated 100% of the time, and one primer (CLU) did not confirm the microarray data.

We next tested gene expression prospectively using RNA from an independent set of 25 NlEpi samples (8 RM and 17 HN; 9 ER+ and 8 ER−) Supplementary Table S1 presents summary information for these samples. All 17 HN samples were tested with each of the six primers (except AHNAK for which 16 HNs were tested) and compared with eight RM samples. Overall, 85 out of 101 (84%) reactions validated the microarray data. All primers validated at about the same rate (76–88%), and no HN sample seemed to be an outlier. Fold changes for each primer's technical and prospective validations were similar (see Figure 2). Summary information for the validations is presented in Supplementary Table S2.

### Gene expression in PM samples

To test whether the gene expression changes in HN NlEpi are also present in the NlEpi of patients at high breast cancer risk, we examined gene expression in 12 PM samples. In six PM samples, we performed microarray analysis and qPCR. In another six PM samples, we performed qPCR only. The ages of PM patients fell within the age range of RM and HN patients (mean PM age=45 years).

**Figure 3:**
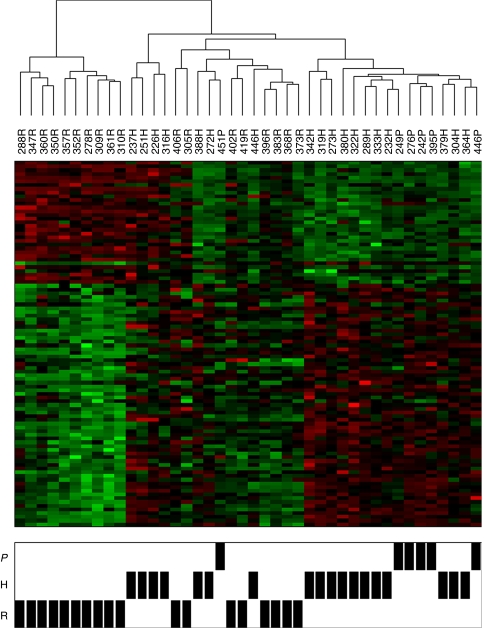
Clustering of RM, HN, and PM samples based on gene expression. Hierarchical clustering of RM, HN, and PM samples using 98 probe sets identified as differentially expressed between 18 RM and 18 HN samples. The relative abundance of each transcript for each sample is represented as a coloured block, with green representing fold changes >2 and red representing probe sets with fold changes <2.

As we had only six PMs on which microarray analysis could be performed, we analysed the gene expression of these six samples using only the 98 probe sets that significantly differed between RM and HN samples. We found that expression of 97 out of 98 (99%) probe sets in PM samples resembled their expression in HN samples: relative to RM expression, the expression of probe sets was in the same direction and approximately of the same magnitude in both PM and HN (Table 2). Further, using these 98 probe sets, analysis of all 42 RM, HN, and PM samples showed that five out of six PM samples clustered with HN, but not with RM samples. The outlier PM clustered in a region composed of both HN and RM samples (Figure 3). In the Monte Carlo procedure, none of the 10 000 simulations produced the same cluster result, hence the probability that our results are due to chance is minimal.

We validated these microarray results using qPCR with primers for the same six genes as in the RM–HN comparison (see Figure 2). With unamplified RNA remaining from the six PM samples and eight of the RM samples used for microarray, we found that 16 out of 21 (76%) reactions validated the microarray data. Each of the four primers (ATF3, BTG2, EGR1, and FOS) validated 100% of the time, and two primers (CLU and AHNAK) did not validate (rates were 60 and 25%, respectively). The validating primers displayed larger fold changes between RM and HN, and between RM and PM (∼3–4-fold), than the non-validating primers (∼two-fold) (see Table 2).

We next tested gene expression prospectively, using NlEpi RNA from an independent set of six PM cases (see Figure 2B). Overall, 21 out of 36 (58%) reactions validated the microarray data. However, validation rates varied not only among primers but also among cases. Four primers (BTG2, EGR1, FOS, and ATF3) validated the microarray data well (rates were 67–100%), and two primers, CLU and AHNAK, did not validate (rates were 33 and 17%, respectively). Among the four validating primers, the mean dCT values differed significantly between the PM and RM groups for BTG2 (*P*=0.03), and approached significance for the others (FOS: *P*=0.08, EGR1: *P*=0.09, ATF3: *P*=0.15). This may reflect a small sample size. Considering these four primers, we examined whether some of the six prospective PM samples validated better than others. Five samples validated well (⩾75%) and one validated poorly (25%). This last sample came from the only patient who underwent PM solely because of family history, she was neither a known BRCA mutation carrier nor did she have a personal history of breast cancer. Summary information for the validations is presented in Supplementary Table S2.

### Analysis and annotation of the 98 probe sets

**Figure 4:**
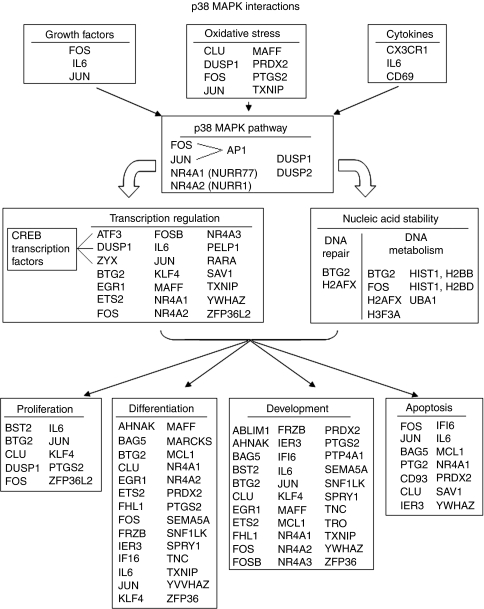
Gene list analysis. Genes identified as participating in the MAPK pathway, MAPK activating functions, and functions induced by MAPK, as annotated in DAVID analysis. Genes are arranged according to the p38 MAPK pathway, with the processes activating the pathway in boxes at the top of the figure, and processes affected by the MAPK pathway in boxes at the bottom of the figure.

The 98 probe-set list was analysed with DAVID, Ingenuity, Panther, and GSEA. All four programmes identified similar functional categories, including cellular metabolism, transcription, stress response, development, apoptosis, and signal transduction. More specifically, DAVID and GSEA identified an overrepresentation of the MAPK signalling pathway using the KEGG pathway annotation component in both analyses. As the 98 probe-set list contained transcription factors specific to p38MAPK, we considered the functions of differentially expressed genes in the context of the p38MAPK pathway (Figure 4).

## Discussion

We aimed to better understand the molecular changes that are present in breast epithelium before clinical or pathological evidence of breast cancer. Therefore, we examined gene expression in 73 snap-frozen microdissected NlEpi samples. We used microarray (and qPCR) in 42 samples (18 RM, 18 HN, and 6 PM), and qPCR alone in 31 independent samples (8 RM, 17 HN, and 6 PM). The microarray analysis identified an age-independent profile consisting of 98 probe sets (corresponding to 86 genes) differentially expressed in the NlEpi of breast cancer cases (HN), compared with controls (RM). Using these 98 probe sets, PM samples clustered with HN and away from RM samples. Prospective validation by qPCR was high (84%) and uniform in the independent HN–RM comparison. Prospective validation was lower on an average (58%), but more heterogeneous among samples, in the independent PM–RM comparison, as might be expected when dealing with cases at variable risk. The 98 probe sets included many transcription factors and were implicated in cancer-related pathways, in particular MAPK. These results suggest that the HN expression profile is not an effect of tumour. Instead, it may be a marker of increased risk of breast cancer development (e.g. field cancerisation), or may reveal some of breast cancer's earliest genomic alterations. Further study of early alterations may identify new preventive agents and risk-assessment tools.

Our findings raise several points for consideration. First, this study differs in fundamental ways from our initial report (Tripathi *et al*, 2008). The sample size is considerably larger. As age influences gene expression (Yau *et al*, 2007; Anders *et al*, 2008; Euhus *et al*, 2008), we tightly age-matched RM and HN subjects, permitting us to identify an age-independent profile. HN samples were balanced for ER+ and ER− tumour status to make our results more generaliseable. We used a novel statistical approach, well suited to a small sample size, to identify differentially expressed genes. We prospectively validated the microarray findings in independent samples. Finally, we examined a rarely studied type of sample – cancer-free breasts from patients undergoing PM because of high breast cancer risk. We are unaware of any earlier reports of gene expression in PMs. Existing papers identify histological and clinical characteristics of PM samples, leading some to advocate for more attention to the genetics of PM (Scott *et al*, 2003; Goldflam *et al*, 2004; Yi *et al*, 2009). Thus, our samples and study design are unique.

Second, despite the important expansions and refinements to the initial study, the RM–HN differences we report here are consistent with those from the earlier study. Even though only 35 (approximately 1 out of 3) probe sets overlap, in both studies, we identify genes belonging to multiple biological and molecular categories, with transcription factors and the p38MAPK pathway apparently overrepresented. This suggests that gene expression differences between the NlEpi of RM and HN samples is a generaliseable finding, applicable to a heterogeneous set of breast cancer samples. Clustering analyses (Supplementary Figures S2 and S3) suggest that the NlEpi of ER+ cases resembles control epithelium (RM) more closely than does the NlEpi of ER− cases.

Investigations of gene expression in HN breast epithelium are limited (Finak *et al*, 2006; Grigoriadis *et al*, 2006; Tripathi *et al*, 2008; Chen *et al*, 2009). Our results contrast with one study that did not find a different gene expression between morphologically normal epithelium microdissected from RM and HN samples (Finak *et al*, 2006). The discrepancy may be explained by differences in study design and analysis. Our results are consistent with those of another study (Grigoriadis *et al*, 2006), although that study had no samples comparable with our HN samples, and with our own earlier findings (Tripathi *et al*, 2008). Another study (Chen *et al*, 2009) found a proliferative gene expression signature in morphologically benign tissue (not necessarily HN) of patients with invasive carcinoma, but no controls were reported.

Third, evaluation of the RM–HN gene list leads to several speculations. One speculation emerges from the observation that the RMs that co-cluster with HNs using the 98-probe-set list are significantly older than the RMs that do not cluster with HNs. As the 36 RM and HN samples were tightly age matched, and all RM cases lacked a personal or strong family history of breast cancer, this age-related clustering of RMs is consistent with the hypothesis that ageing itself is associated with genomic changes resembling changes of early cancer. Another speculation relates to the function of transcription factors. We found multiple transcription factors, the expression of which decreased in HN samples (e.g. *ATF3*, *MAFF*, and *TXNIP*). Transcription factors may be preferentially regulated by methylation (Bloushtain-Qimron *et al*, 2008) and methylation (and other epigenetic events) is believed to contribute to the early stages of carcinogenesis (Tommasi *et al*, 2009). Thus, early epigenetic events could lead to the decreased expression of transcription factors that we see in HN. These epigenetic changes may in some manner determine the subtype of tumour that may arise from a particular cell or TDLU. A final speculation relates to the potential function of the p38MAPK pathway. We found that MAPK pathway gene expression was decreased in HN compared with RM samples, but was less decreased in PM samples. Thus, MAPK pathway deregulation may be implicated early in breast cancer development, and may differentiate PM from HN epithelium. The MAPK functions in cell cycle and transcriptional regulation and in the immune response may thus inhibit tumourigenesis (Bradham and McClay, 2006; Cuenda and Rousseau, 2007), Therefore, it is not surprising to see a higher expression of genes in this pathway in NlEpi from women without breast cancer (RM and perhaps PM samples). Alternatively, a decreased expression of MAPK in the epithelium may reflect signals arising from the microenvironment surrounding the epithelium. If so, it is consistent with the view that the microenvironment has a crucial function in suppressing malignant transformation or behaviour (Spencer *et al*, 2007; Hu and Polyak, 2008).

Finally, our analysis of PM gene expression shows that, in general, PM samples resemble HN rather than RM samples. As the PM breast does not contain cancer, the HN-like changes cannot be an effect of the tumour. Instead, they may be a marker of increased breast cancer risk – the concept of mammary field cancerisation is longstanding, and has recently been reviewed (Heaphy *et al*, 2009). Alternatively, the HN-like changes could reflect breast cancer's earliest gene expression changes. The variable validation rate among PM samples would be expected in a heterogeneous group composed of high-risk women. Low validation of specific genes could reflect splice variants. If future studies confirm these findings, then evaluation of gene expression in NlEpi could improve risk assessment and affect clinical decision making with regard to this controversial procedure (Borgen *et al*, 1998; Hartmann *et al*, 1999, 2004; Eisinger, 2007; Giuliano *et al*, 2007; Briasoulis and Roukos, 2008). These findings could also identify new prevention agents, by finding drugs or interventions that modify or reverse this transcriptional signature.

The primary limitation of our study is the sample size, which is relatively small because of the nature of our samples: fresh, microdissected primary epithelium from untreated women. We have made every effort to counterbalance this limitation by using a statistical analysis suitable for small sample sizes and by using novel and important samples that provide accurate *in vivo* data.

## Conclusions

We find that a distinct profile distinguishes the NlEpi of breast cancer cases (HN), including both ER+ and ER− cancers, from that of controls (RM), and this profile can be discerned in PM. This suggests that the HN profile is not an effect of the tumour, but instead may be a marker of increased breast cancer risk, or a reflection of breast cancer's earliest gene expression changes. Gene expression changes before histological abnormalities could be used to elucidate pathways altered early in breast carcinogenesis and to develop novel risk-assessment tools, prevention agents, and therapies.

## Figures and Tables

**Table 1:** Characteristics of 42 cases whose NlEpi was analysed by microarray

**HN**					**RM**		**PM**		
**Sample**	**Age (years)**	**Distance from tumour**	**ER/PR/HER2^a^**	**Stage^b^**	**Sample**	**Age (years)**	**Sample**	**Age (years)**	**Breast cancer history^c^**
319H	34	<2 cm	−/−/−	IIIA	360R^d^	36	395P	35	+
316H^d^	39	<2 cm	+/−/−	IIA	352R^d^	41	276P	36	−
379H	43	<2 cm	+/+/+	I	347R^d^	43	451P	43	+
251H^d^	45	<2 cm	+/+/−	0	278R^d^	44	242P	46	+
289H	47	<2 cm	−/−/−	IIA	373R	44	446AP	54	+
364H	47	>2 cm	−/−/+	IIIA	350R^d^	47	249P	57	−
342H	48	<2 cm	−/−/−	IIA	309R^d^	49			
304BH^d^	49	<2 cm	+/+/+	I	357R^d^	49			
273H	49	<2 cm	−/−/+	III	368R^d^	49			
380H	53	<2 cm	+/+/+	IIA	288R^d^	52			
446BH	54	>2 cm	+/+/−	IIIA	402R	55			
237H^d^	55	?	+/−/−	I	383R	55			
322H	58	<2 cm	−/−/−	I	406R	56			
272H	58	>2 cm	−/−/+	IIIC	396R	57			
388AH	58	<2 cm	+/−/−	I	361R^d^	57			
232H	59	>2 cm	+/+/na	0	419R	57			
226H^d^	61	<2 cm	−/−/na	IIIA	310R^d^	60			
333H	76	>2 cm	−/−/+	IIB	305R	75			

Abbreviations: ER=oestrogen receptor; HER2=human epidermal growth factor receptor-2; HN=histologically normal; PM=prophylactic mastectomy; PR=progesterone receptor; NlEpi=histologically normal breast epithelium; RM=reduction mammoplasty.

a na=not available.

b Stage using AJCC stage criteria.

c +=previous breast cancer in the contralateral breast, but no history of ipsilateral cancer. 451P is a BRCA2 mutation carrier.

d Used in Tripathi *et al* (2008).

**Table 2:** Comparison of gene expression fold change for 98 probe sets: RM : HN and RM : PM

**Symbol^a^**	**RM : HN fold^b^**	**Probability score^c^**	***P*-value^d^**	**RM : PM fold^e^**	**Symbol^a^**	**RM : HN fold^b^**	**Probability score^c^**	***P*-value^d^**	**RM : PM fold^e^**
ATF3^a^	5.72	1.52E−07	1.22E−06	3.39	NEDD4L	2.11	8.35E−05	1.15E−04	2.13
FOS^a^	4.62	1.23E−04	2.69E−05	2.81	ARL4C	2.11	4.00E−05	3.43E−04	1.83
FOSB^a^	4.32	6.33E−04	7.99E−05	1.76	AQP3	2.11	1.46E−03	1.01E−02	2.15
NR4A2^a^	4.02	5.80E−07	4.57E−07	3.21	AHNAK	2.11	2.59E−04	1.54E−03	1.74
HIST1H2BG	3.99	5.15E−04	1.99E−01	4.40	LEPROT	2.10	1.05E−03	1.93E−03	3.10
CDH19^a^	3.94	2.45E−04	1.73E−02	1.66	MCL1	2.10	2.20E−04	6.17E−04	1.98
HIST1H2BB^a^	3.85	7.53E−09	2.43E−08	4.21	CHN1	2.10	9.45E−04	5.86E−03	1.67
NR4A2^a^	3.83	2.76E−06	1.09E−05	3.43	SEMA5A	2.10	6.21E−04	4.38E−03	1.86
IER2^a^	3.77	1.08E−08	1.82E−08	3.52	RGS5	2.10	1.02E−03	3.52E−03	2.75
BTG2^a^	3.69	3.04E−07	2.15E−06	3.92	IER3	2.09	3.00E−04	6.81E−04	1.76
NA^a^	3.68	1.37E−05	1.62E−05	4.57	TIMM23	2.08	8.16E−06	1.41E−05	2.29
NR4A2^a^	3.46	6.02E−07	1.34E−06	3.33	NSF	2.08	5.52E−04	7.69E−03	2.50
EIF1^a^	3.27	1.18E−07	5.47E−07	2.92	PTP4A1^a^	2.08	6.24E−04	3.98E−03	1.81
JUN^a^	3.26	1.32E−08	6.70E−09	1.87	FHL1	2.08	1.50E−03	3.17E−02	1.46
ZFAND5	3.10	2.37E−05	1.21E−03	4.03	BAG5	2.08	6.01E−04	6.49E−03	2.29
NA^a^	2.92	3.15E−05	2.44E−04	2.13	EIF5^a^	2.05	5.40E−05	4.01E−04	1.97
DUSP1^a^	2.87	1.07E−05	4.37E−06	2.17	NIPBL	2.04	1.01E−04	3.74E−04	1.74
NR4A3^a^	2.85	3.72E−06	3.48E−05	3.07	GMPPA	0.48	6.45E−04	8.71E−03	0.58
CD69^a^	2.84	1.38E−04	8.07E−04	4.85	TRO^a^	0.48	1.27E−03	8.30E−04	0.37
SPRY1	2.70	3.26E−04	2.37E−03	3.19	GNB2	0.48	2.13E−04	1.04E−03	0.49
IL6	2.66	5.84E−04	7.09E−03	2.56	CST3	0.47	1.49E−03	7.13E−03	0.41
PTP4A1^a^	2.65	2.12E−07	1.01E−06	2.69	PDXDC2	0.47	8.07E−04	2.90E−03	0.35
TRIM37	2.60	3.61E−04	3.88E−03	3.63	ZYX	0.47	3.92E−04	7.60E−04	0.38
RGS2^a^	2.60	1.24E−03	1.28E−03	1.37	UBA1	0.47	1.32E−03	3.54E−03	0.41
KLF4^a^	2.59	2.54E−04	6.69E−04	1.56	MARCKS	0.47	1.41E−03	2.02E−02	0.35
CX3CR1	2.58	1.69E−04	2.14E−02	2.65	RARA	0.46	3.51E−04	5.48E−04	0.47
NR4A1^a^	2.56	4.63E−04	7.42E−04	1.98	PELP1	0.46	2.85E−05	8.64E−05	0.48
H3F3B^a^	2.53	1.04E−07	7.24E−08	1.93	PRDX2	0.46	1.32E−03	6.95E−04	0.41
DUSP2^a^	2.53	4.35E−04	4.81E−03	1.25	RPL28	0.45	1.35E−04	1.47E−04	0.42
TXNIP	2.50	3.17E−04	5.90E−04	2.96	COL16A1	0.44	1.41E−03	9.05E−04	0.40
PTGS2^a^	2.47	7.16E−04	3.11E−03	1.87	BAT2	0.44	4.61E−04	1.46E−02	0.45
EGR1^a^	2.47	3.83E−04	2.34E−04	3.64	PLTP	0.44	5.35E−04	1.65E−03	0.39
SNF1LK^a^	2.37	2.84E−05	4.52E−05	2.55	CLU	0.44	2.13E−04	5.33E−04	0.52
EGR1^a^	2.37	7.45E−05	4.98E−05	2.63	PIB5PA	0.43	3.66E−04	1.21E−04	0.35
PTP4A1^a^	2.34	2.92E−05	7.13E−04	2.62	TNC	0.42	4.90E−04	6.46E−03	0.56
CD93	2.33	2.54E−04	4.60E−03	2.58	IFI6	0.42	2.74E−04	5.88E−03	0.65
MAFF	2.31	5.29E−04	1.04E−03	2.86	INTS3	0.42	6.59E−04	9.70E−04	0.46
SAV1	2.29	6.26E−04	1.00E−02	2.94	H2AFX	0.42	2.56E−04	4.63E−04	0.41
EIF4A1^a^	2.28	2.92E−06	9.00E−06	2.38	FLJ11292	0.42	9.81E−04	1.83E−01	0.71
JUN^a^	2.25	3.43E−08	7.17E−08	3.39	RPS26	0.42	9.31E−04	1.40E−03	0.36
ZFP36^a^	2.23	1.29E−04	4.28E−04	2.04	IFI35	0.40	7.07E−04	4.10E−04	0.43
RGS5	2.23	1.34E−03	1.97E−03	2.49	ZFP36L2	0.40	1.38E−03	5.99E−02	0.45
YWHAZ^a^	2.22	5.60E−04	4.32E−03	1.93	C17orf101	0.38	3.20E−04	4.73E−04	0.39
PNMAL1	2.20	1.05E−03	1.18E−02	1.88	LRRC14	0.37	1.08E−03	9.10E−04	0.32
JUN^a^	2.19	2.34E−05	1.20E−05	2.13	NPIPL3	0.34	1.10E−03	3.59E−03	0.78
MCL1^a^	2.17	5.52E−05	3.24E−04	2.47	BST2	0.31	9.68E−05	9.65E−03	0.33
FRZB	2.15	1.36E−03	2.02E−02	2.44	SEC14L1	0.30	4.14E−05	6.00E−03	0.42
ITGBL1	2.15	1.67E−03	2.23E−02	1.85	CALD1	0.27	7.01E−06	5.90E−05	0.21
ABLIM1	2.13	7.96E−05	1.29E−04	2.07	CSN2	0.10	9.09E−05	1.52E−01	1.30

Abbreviations: BADGE=Bayesian Analysis of Differential Gene Expression; HN=histologically normal; PM=prophylactic mastectomy; RM=reduction mammoplasty.

a Identified in Tripathi *et al* (2008).

b Fold change calculated in BADGE. The sample type on the right is the reference (e.g. ATF3 is overexpressed in RM compared to HN).

c Probability score calculated in BADGE.

d *P*-values from two-sided *t*-test using log-transformed data.

e Fold change calculated by dividing mean RM expression value by mean PM expression value.

## References

[bib1] Allred DC, Wu Y, Mao S, Nagtegaal ID, Lee S, Perou CM, Mohsin SK, O’Connell P, Tsimelzon A, Medina D (2008) Ductal carcinoma *in situ* and the emergence of diversity during breast cancer evolution. Clin Cancer Res 14: 370–378 18223211 10.1158/1078-0432.CCR-07-1127

[bib2] Anders CK, Acharya CR, Hsu DS, Broadwater G, Garman K, Foekens JA, Zhang Y, Wang Y, Marcom K, Marks JR, Mukherjee S, Nevins JR, Blackwell KL, Potti A (2008) Age-specific differences in oncogenic pathway deregulation seen in human breast tumors. PLoS One 3: e1373 18167534 10.1371/journal.pone.0001373 PMC2148101

[bib3] Bao T, Davidson NE (2008) Gene expression profiling of breast cancer. Adv Surg 42: 249–260 18953822 10.1016/j.yasu.2008.03.002 PMC2775529

[bib4] Bloushtain-Qimron N, Yao J, Snyder EL, Shipitsin M, Campbell LL, Mani SA, Hu M, Chen H, Ustyansky V, Antosiewicz JE, Argani P, Halushka MK, Thomson JA, Pharoah P, Porgador A, Sukumar S, Parsons R, Richardson AL, Stampfer MR, Gelman RS, Nikolskaya T, Nikolsky Y, Polyak K (2008) Cell type-specific DNA methylation patterns in the human breast. Proc Natl Acad Sci USA 105: 14076–14081 18780791 10.1073/pnas.0805206105 PMC2532972

[bib5] Borgen PI, Hill AD, Tran KN, Van Zee KJ, Massie MJ, Payne D, Biggs CG (1998) Patient regrets after bilateral prophylactic mastectomy. Ann Surg Oncol 5: 603–606 9831108 10.1007/BF02303829

[bib6] Bradham C, McClay DR (2006) p38 MAPK in development and cancer. Cell Cycle 5: 824–828 16627995 10.4161/cc.5.8.2685

[bib7] Briasoulis E, Roukos DH (2008) Contralateral prophylactic mastectomy: mind the genetics. J Clin Oncol 26: 1909–1910; author reply 1910 18398162 10.1200/JCO.2008.16.0275

[bib8] Calza S, Hall P, Auer G, Bjohle J, Klaar S, Kronenwett U, Liu ET, Miller L, Ploner A, Smeds J, Bergh J, Pawitan Y (2006) Intrinsic molecular signature of breast cancer in a population-based cohort of 412 patients. Breast Cancer Res 8: R34 16846532 10.1186/bcr1517 PMC1779468

[bib9] Chen DT, Nasir A, Culhane A, Venkataramu C, Fulp W, Rubio R, Wang T, Agrawal D, McCarthy SM, Gruidl M, Bloom G, Anderson T, White J, Quackenbush J, Yeatman T (2009) Proliferative genes dominate malignancy-risk gene signature in histologically-normal breast tissue. Breast Cancer Res Treat 119: 335–346 19266279 10.1007/s10549-009-0344-y PMC2796276

[bib10] Cuenda A, Rousseau S (2007) p38 MAP-kinases pathway regulation, function and role in human diseases. Biochim Biophys Acta 1773: 1358–1375 17481747 10.1016/j.bbamcr.2007.03.010

[bib11] Eisinger F (2007) Prophylactic mastectomy: ethical issues. Br Med Bull 81–82: 7–19 10.1093/bmb/ldm003 17409120

[bib12] Euhus DM, Bu D, Milchgrub S, Xie XJ, Bian A, Leitch AM, Lewis CM (2008) DNA methylation in benign breast epithelium in relation to age and breast cancer risk. Cancer Epidemiol Biomarkers Prev 17: 1051–1059 18483325 10.1158/1055-9965.EPI-07-2582

[bib13] Finak G, Sadekova S, Pepin F, Hallett M, Meterissian S, Halwani F, Khetani K, Souleimanova M, Zabolotny B, Omeroglu A, Park M (2006) Gene expression signatures of morphologically normal breast tissue identify basal-like tumors. Breast Cancer Res 8: R58 17054791 10.1186/bcr1608 PMC1779486

[bib14] Gail MH, Brinton LA, Byar DP, Corle DK, Green SB, Schairer C, Mulvihill JJ (1989) Projecting individualized probabilities of developing breast cancer for white females who are being examined annually. J Natl Cancer Inst 81: 1879–1886 2593165 10.1093/jnci/81.24.1879

[bib15] Gail MH, Costantino JP, Pee D, Bondy M, Newman L, Selvan M, Anderson GL, Malone KE, Marchbanks PA, McCaskill-Stevens W, Norman SA, Simon MS, Spirtas R, Ursin G, Bernstein L (2007) Projecting individualized absolute invasive breast cancer risk in African American women. J Natl Cancer Inst 99: 1782–1792 18042936 10.1093/jnci/djm223

[bib16] Giuliano AE, Boolbol S, Degnim A, Kuerer H, Leitch AM, Morrow M (2007) Society of Surgical Oncology: position statement on prophylactic mastectomy. Approved by the Society of Surgical Oncology Executive Council, March 2007. Ann Surg Oncol 14: 2425–2427 17597344 10.1245/s10434-007-9447-z

[bib17] Goldflam K, Hunt KK, Gershenwald JE, Singletary SE, Mirza N, Kuerer HM, Babiera GV, Ames FC, Ross MI, Feig BW, Sahin AA, Arun B, Meric-Bernstam F (2004) Contralateral prophylactic mastectomy. Predictors of significant histologic findings. Cancer 101: 1977–1986 15389473 10.1002/cncr.20617

[bib18] Grigoriadis A, Mackay A, Reis-Filho JS, Steele D, Iseli C, Stevenson BJ, Jongeneel CV, Valgeirsson H, Fenwick K, Iravani M, Leao M, Simpson AJ, Strausberg RL, Jat PS, Ashworth A, Neville AM, O’Hare MJ (2006) Establishment of the epithelial-specific transcriptome of normal and malignant human breast cells based on MPSS and array expression data. Breast Cancer Res 8: R56 17014703 10.1186/bcr1604 PMC1779497

[bib19] Hartmann LC, Degnim A, Schaid DJ (2004) Prophylactic mastectomy for BRCA1/2 carriers: progress and more questions. J Clin Oncol 22: 981–983 14981099 10.1200/JCO.2004.01.925

[bib20] Hartmann LC, Schaid DJ, Woods JE, Crotty TP, Myers JL, Arnold PG, Petty PM, Sellers TA, Johnson JL, McDonnell SK, Frost MH, Jenkins RB (1999) Efficacy of bilateral prophylactic mastectomy in women with a family history of breast cancer. N Engl J Med 340: 77–84 9887158 10.1056/NEJM199901143400201

[bib21] Heaphy CM, Griffith JK, Bisoffi M (2009) Mammary field cancerization: molecular evidence and clinical importance. Breast Cancer Res Treat 118: 229–239 19685287 10.1007/s10549-009-0504-0

[bib22] Hu M, Polyak K (2008) Molecular characterisation of the tumour microenvironment in breast cancer. Eur J Cancer 44: 2760–2765 19026532 10.1016/j.ejca.2008.09.038 PMC2729518

[bib23] Kapp AV, Jeffrey SS, Langerod A, Borresen-Dale AL, Han W, Noh DY, Bukholm IR, Nicolau M, Brown PO, Tibshirani R (2006) Discovery and validation of breast cancer subtypes. BMC Genomics 7: 231 16965636 10.1186/1471-2164-7-231 PMC1574316

[bib24] King C, Guo N, Frampton GM, Gerry NP, Lenburg ME, Rosenberg CL (2005) Reliability and reproducibility of gene expression measurements using amplified RNA from laser-microdissected primary breast tissue with oligonucleotide arrays. J Mol Diagn 7: 57–64 15681475 10.1016/S1525-1578(10)60009-8 PMC1867505

[bib25] Kuerer HM, Albarracin CT, Yang WT, Cardiff RD, Brewster AM, Symmans WF, Hylton NM, Middleton LP, Krishnamurthy S, Perkins GH, Babiera G, Edgerton ME, Czerniecki BJ, Arun BK, Hortobagyi GN (2009) Ductal carcinoma *in situ*: state of the science and roadmap to advance the field. J Clin Oncol 27: 279–288 19064970 10.1200/JCO.2008.18.3103

[bib26] Ma XJ, Hilsenbeck SG, Wang W, Ding L, Sgroi DC, Bender RA, Osborne CK, Allred DC, Erlander MG (2006) The HOXB13:IL17BR expression index is a prognostic factor in early-stage breast cancer. J Clin Oncol 24: 4611–4619 17008703 10.1200/JCO.2006.06.6944

[bib27] Mastracci TL, Boulos FI, Andrulis IL, Lam WL (2007) Genomics and premalignant breast lesions: clues to the development and progression of lobular breast cancer. Breast Cancer Res 9: 215 18036272 10.1186/bcr1785 PMC2246168

[bib28] Mootha VK, Lindgren CM, Eriksson KF, Subramanian A, Sihag S, Lehar J, Puigserver P, Carlsson E, Ridderstrale M, Laurila E, Houstis N, Daly MJ, Patterson N, Mesirov JP, Golub TR, Tamayo P, Spiegelman B, Lander ES, Hirschhorn JN, Altshuler D, Groop LC (2003) PGC-1alpha-responsive genes involved in oxidative phosphorylation are coordinately downregulated in human diabetes. Nat Genet 34: 267–273 12808457 10.1038/ng1180

[bib29] Paik S, Shak S, Tang G, Kim C, Baker J, Cronin M, Baehner FL, Walker MG, Watson D, Park T, Hiller W, Fisher ER, Wickerham DL, Bryant J, Wolmark N (2004) A multigene assay to predict recurrence of tamoxifen-treated, node-negative breast cancer. N Engl J Med 351: 2817–2826 15591335 10.1056/NEJMoa041588

[bib30] Perou CM, Sorlie T, Eisen MB, van de Rijn M, Jeffrey SS, Rees CA, Pollack JR, Ross DT, Johnsen H, Akslen LA, Fluge O, Pergamenschikov A, Williams C, Zhu SX, Lonning PE, Borresen-Dale AL, Brown PO, Botstein D (2000) Molecular portraits of human breast tumours. Nature 406: 747–752 10963602 10.1038/35021093

[bib31] Scott CI, Iorgulescu DG, Thorne HJ, Henderson MA, Phillips KA (2003) Clinical, pathological and genetic features of women at high familial risk of breast cancer undergoing prophylactic mastectomy. Clin Genet 64: 111–121 12859406 10.1034/j.1399-0004.2003.00097.x

[bib32] Sebastiani P, Xe H, Ramoni MF (2006) Bayesian analysis of comparative microarray experiments by model averaging. Bayesian Anal J 1: 707–732

[bib33] Simpson PT, Reis-Filho JS, Gale T, Lakhani SR (2005) Molecular evolution of breast cancer. J Pathol 205: 248–254 15641021 10.1002/path.1691

[bib34] Singh D, Febbo PG, Ross K, Jackson DG, Manola J, Ladd C, Tamayo P, Renshaw AA, D’Amico AV, Richie JP, Lander ES, Loda M, Kantoff PW, Golub TR, Sellers WR (2002) Gene expression correlates of clinical prostate cancer behavior. Cancer Cell 1: 203–209 12086878 10.1016/s1535-6108(02)00030-2

[bib35] Sorlie T, Perou CM, Tibshirani R, Aas T, Geisler S, Johnsen H, Hastie T, Eisen MB, van de Rijn M, Jeffrey SS, Thorsen T, Quist H, Matese JC, Brown PO, Botstein D, Eystein Lonning P, Borresen-Dale AL (2001) Gene expression patterns of breast carcinomas distinguish tumor subclasses with clinical implications. Proc Natl Acad Sci USA 98: 10869–10874 11553815 10.1073/pnas.191367098 PMC58566

[bib36] Sorlie T, Tibshirani R, Parker J, Hastie T, Marron JS, Nobel A, Deng S, Johnsen H, Pesich R, Geisler S, Demeter J, Perou CM, Lonning PE, Brown PO, Borresen-Dale AL, Botstein D (2003) Repeated observation of breast tumor subtypes in independent gene expression data sets. Proc Natl Acad Sci USA 100: 8418–8423 12829800 10.1073/pnas.0932692100 PMC166244

[bib37] Sotiriou C, Neo SY, McShane LM, Korn EL, Long PM, Jazaeri A, Martiat P, Fox SB, Harris AL, Liu ET (2003) Breast cancer classification and prognosis based on gene expression profiles from a population-based study. Proc Natl Acad Sci USA 100: 10393–10398 12917485 10.1073/pnas.1732912100 PMC193572

[bib38] Sotiriou C, Pusztai L (2009) Gene-expression signatures in breast cancer. N Engl J Med 360: 790–800 19228622 10.1056/NEJMra0801289

[bib39] Spencer VA, Xu R, Bissell MJ (2007) Extracellular matrix, nuclear and chromatin structure, and gene expression in normal tissues and malignant tumors: a work in progress. Adv Cancer Res 97: 275–294 17419950 10.1016/S0065-230X(06)97012-2 PMC2912285

[bib40] Subramanian A, Tamayo P, Mootha VK, Mukherjee S, Ebert BL, Gillette MA, Paulovich A, Pomeroy SL, Golub TR, Lander ES, Mesirov JP (2005) Gene set enrichment analysis: a knowledge-based approach for interpreting genome-wide expression profiles. Proc Natl Acad Sci USA 102: 15545–15550 16199517 10.1073/pnas.0506580102 PMC1239896

[bib41] Tamimi RM, Baer HJ, Marotti J, Galan M, Galaburda L, Fu Y, Deitz AC, Connolly JL, Schnitt SJ, Colditz GA, Collins LC (2008) Comparison of molecular phenotypes of ductal carcinoma *in situ* and invasive breast cancer. Breast Cancer Res 10: R67 18681955 10.1186/bcr2128 PMC2575540

[bib42] Thompson A, Brennan K, Cox A, Gee J, Harcourt D, Harris A, Harvie M, Holen I, Howell A, Nicholson R, Steel M, Streuli C (2008) Evaluation of the current knowledge limitations in breast cancer research: a gap analysis. Breast Cancer Res 10: R26 18371194 10.1186/bcr1983 PMC2397525

[bib43] Tommasi S, Karm DL, Wu X, Yen Y, Pfeifer GP (2009) Methylation of homeobox genes is a frequent and early epigenetic event in breast cancer. Breast Cancer Res 11: R14 19250546 10.1186/bcr2233 PMC2687719

[bib44] Tripathi A, King C, de la Morenas A, Perry VK, Burke B, Antoine GA, Hirsch EF, Kavanah M, Mendez J, Stone M, Gerry NP, Lenburg ME, Rosenberg CL (2008) Gene expression abnormalities in histologically normal breast epithelium of breast cancer patients. Int J Cancer 122: 1557–1566 18058819 10.1002/ijc.23267

[bib45] van ‘t Veer LJ, Dai H, van de Vijver MJ, He YD, Hart AA, Mao M, Peterse HL, van der Kooy K, Marton MJ, Witteveen AT, Schreiber GJ, Kerkhoven RM, Roberts C, Linsley PS, Bernards R, Friend SH (2002) Gene expression profiling predicts clinical outcome of breast cancer. Nature 415: 530–536 11823860 10.1038/415530a

[bib46] van de Vijver MJ, He YD, van’t Veer LJ, Dai H, Hart AA, Voskuil DW, Schreiber GJ, Peterse JL, Roberts C, Marton MJ, Parrish M, Atsma D, Witteveen A, Glas A, Delahaye L, van der Velde T, Bartelink H, Rodenhuis S, Rutgers ET, Friend SH, Bernards R (2002) A gene-expression signature as a predictor of survival in breast cancer. N Engl J Med 347: 1999–2009 12490681 10.1056/NEJMoa021967

[bib47] Yang H, Crawford N, Lukes L, Finney R, Lancaster M, Hunter KW (2005) Metastasis predictive signature profiles pre-exist in normal tissues. Clin Exp Metastasis 22: 593–603 16475030 10.1007/s10585-005-6244-6 PMC2048974

[bib48] Yau C, Fedele V, Roydasgupta R, Fridlyand J, Hubbard A, Gray JW, Chew K, Dairkee SH, Moore DH, Schittulli F, Tommasi S, Paradiso A, Albertson DG, Benz CC (2007) Aging impacts transcriptomes but not genomes of hormone-dependent breast cancers. Breast Cancer Res 9: R59 17850661 10.1186/bcr1765 PMC2216076

[bib49] Yi M, Meric-Bernstam F, Middleton LP, Arun BK, Bedrosian I, Babiera GV, Hwang RF, Kuerer HM, Yang W, Hunt KK (2009) Predictors of contralateral breast cancer in patients with unilateral breast cancer undergoing contralateral prophylactic mastectomy. Cancer 115: 962–971 19172584 10.1002/cncr.24129 PMC4365476

