# Supplementary figures and images for: Gene expression in histologically normal epithelium from breast cancer patients and from cancer-free prophylactic mastectomy patients shares a similar profile
Source: Br J Cancer. 2010 Mar 2;102(8):1284–93. doi: 10.1038/sj.bjc.6605576 (PMC2855998; doi:10.1038/sj.bjc.6605576)

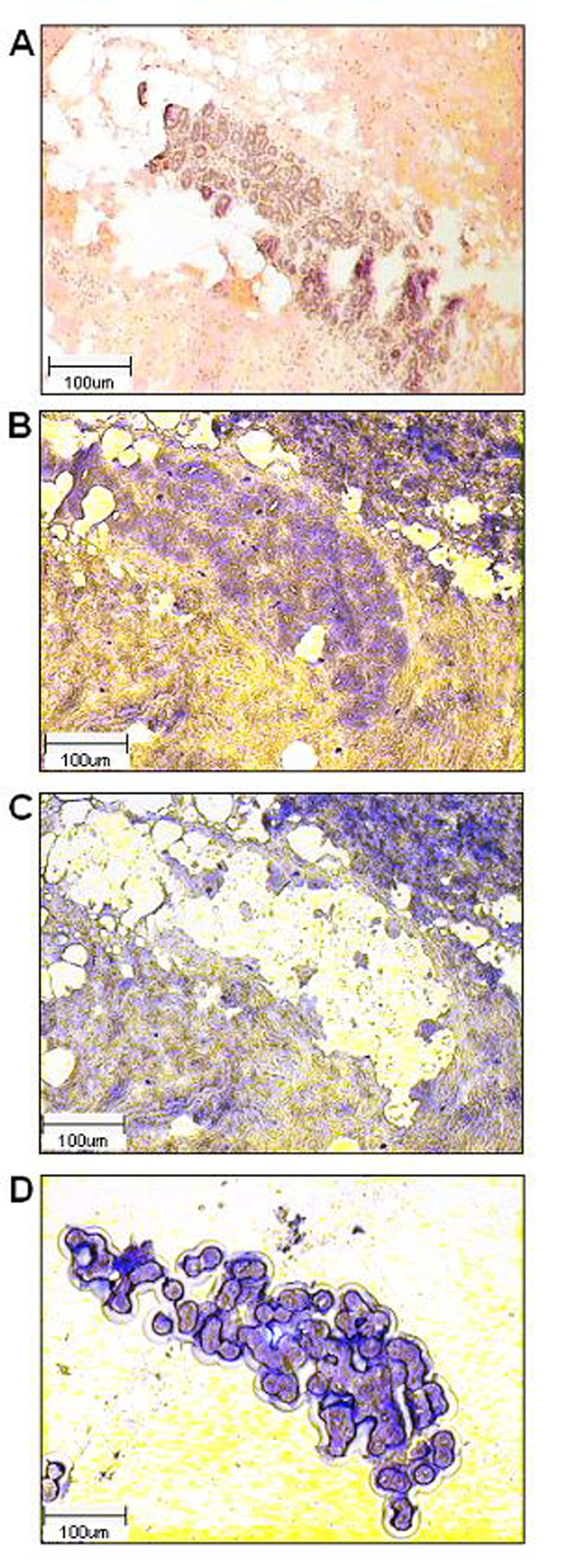

Supplement: Supplementary Figure S1 [file 6605576x1.tif]

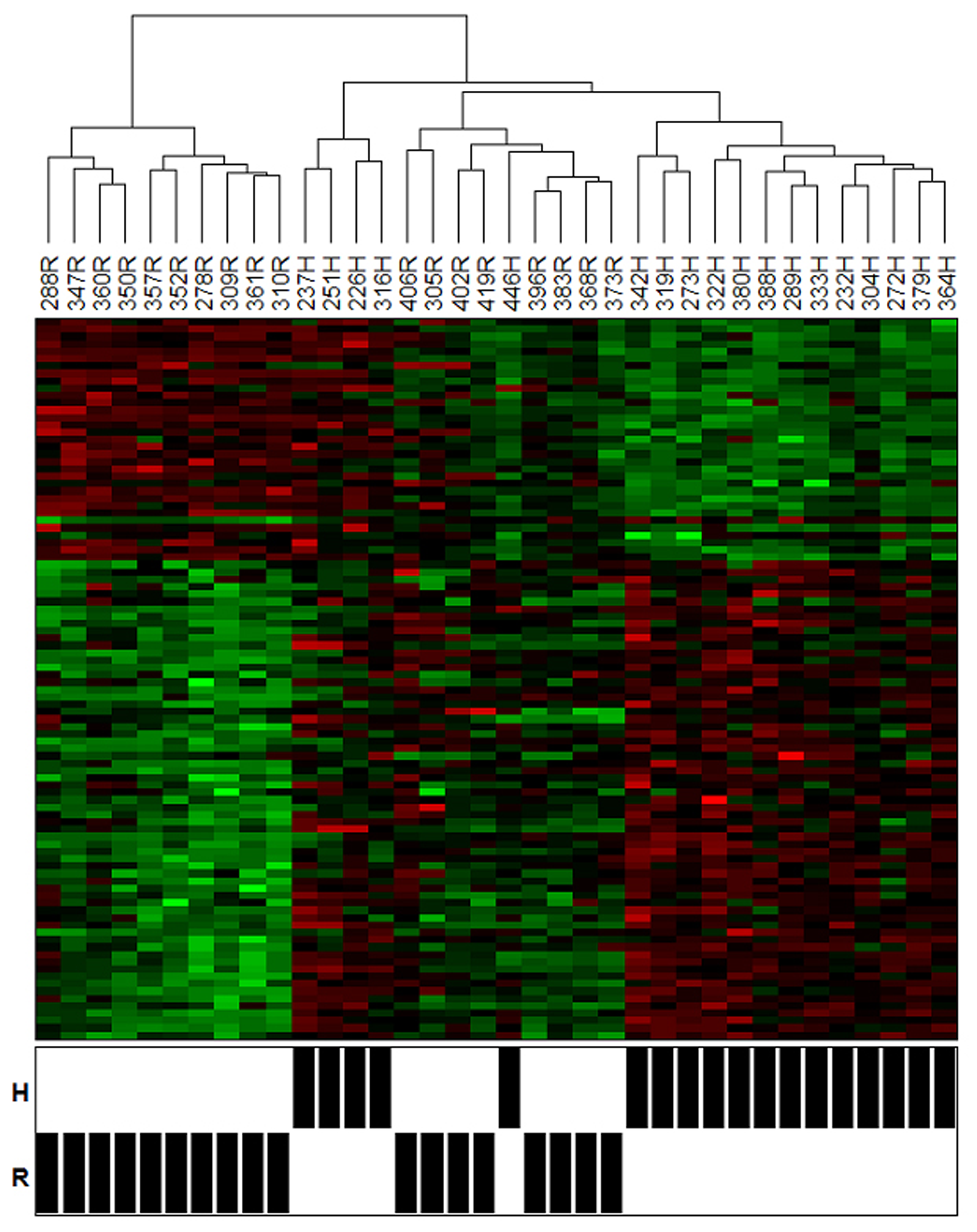

Supplement: Supplementary Figure S2 [file 6605576x2.tif]

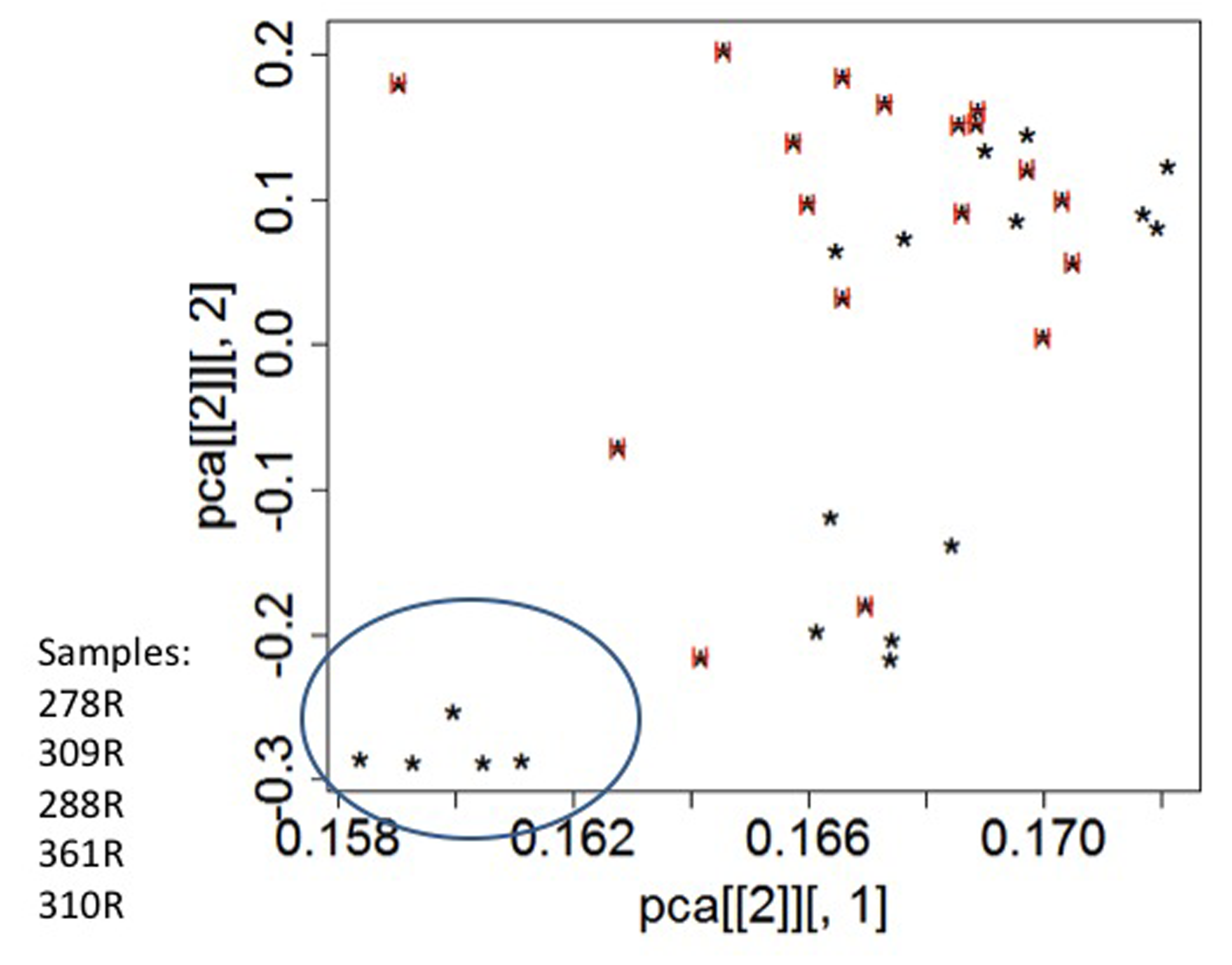

Supplement: Supplementary Figure S3 [file 6605576x3.tif]
