# Supplementary material for: Gene expression in histologically normal epithelium from breast cancer patients and from cancer-free prophylactic mastectomy patients shares a similar profile
Source: Br J Cancer. 2010 Mar 2;102(8):1284–93. doi: 10.1038/sj.bjc.6605576 (PMC2855998; doi:10.1038/sj.bjc.6605576)
Supplement: Supplementary Figure Legends [file 6605576x4.doc]

**Supplemental File Legends**

**Figure S1. Microdissection of Histologically Normal Epithelium**

**A.** A TDLU from a 10um guide section stained with hematoxylin and eosin (H&E). **B.** A 10um section prepared for laser capture microdissection with a lighter H&E stain. **C.** The same section as B, post-dissection. **D.** A view of the cap post-dissection. All slides are 10X magnification.

**Figure S2. Clustering of RM and HN Samples**

Hierarchical clustering of RM and HN samples using the 98 probesets identified as differentially expressed between the 18 RM and 18 HN samples. The relative abundance of each transcript for each sample is represented as a colored block, with green representing fold changes greater than 2 and red representing fold changes less than 2.

**Figure S3. Principal Components Analysis of RM and HN Samples**

Principal components analysis of the 12962 probesets expressed in at least 20% of the 18 RM and 18 HN samples. One cluster of 5 RM samples is seen. These 5 RM samples cluster on the far left side of the hierarchical clustering analysis (**Figure S2**) performed on the same 36 samples using just the 98 probesets.
